# Clinical characteristics and prognostic factor in juvenile dermatomyositis: data of the Spanish registry

**DOI:** 10.1186/s12969-024-00999-9

**Published:** 2024-07-22

**Authors:** Sonia Carriquí-Arenas, Juan Manuel Mosquera, Estefanía Quesada-Masachs, Mireia López, Daniel Clemente, Alina Boteanu, Clara Udaondo, Jaime de Inocencio, Juan Carlos Nieto, Leyre Riancho, Esmeralda Núñez, Judith Sánchez-Manubens, María José Lirola, Rosa Roldán, Marisol Camacho, Melania Martínez, Marta Medrano, Paula Alcañiz, Jordi Antón, Estíbaliz Iglesias

**Affiliations:** 1 Pediatric Rheumatology Department, Hospital Sant Joan de Déu, Barcelona, Spain; 2 Institut de Recerca Sant Joan de Déu, Barcelona, Spain; 3 Rheumatology Department, Pediatric Rheumatology Unit, Vall d’Hebron University Hospital, Barcelona Hospital Campus, Barcelona, Spain; 4 Pediatric Rheumatology Unit, Niño Jesús Hospital, Madrid, Spain; 5 Rheumatology Department, Ramón y Cajal University Hospital, Madrid, Spain; 6 Pediatric Rheumatology Unit, La Paz University Children’s Hospital, CIBERINFEC (CIBER research network, Carlos III research institute), Madrid, Spain; 7 Pediatric Rheumatology Unit, University Hospital 12 de octubre, Madrid, Spain; 8 Pediatrics Complutense, University of Madrid, Madrid, Spain; 9 Rheumatology Department, Gregorio Marañón General University Hospital, Madrid, Spain; 10 Rheumatology Department, Valdecilla Hospital, Santander, Spain; 11 Pediatric Rheumatology Unit, Pediatric Unit. Maternal and Child Hospital, Regional University Hospital of Malaga, Málaga, Spain; 12 Pediatric Rheumatology Unit, Pediatric Department, Parc Taulí University Hospital, Sabadell, Spain; 13 Investigation and innovation Institute I3PT, University Autónoma, Barcelona, Spain; 14 Pediatric, Universtiy Hospital Macarena. IHP Group (Hispalense Institute of Pediatrics), Sevilla, Spain; 15 Pediatric Rheumatology Unit, Rheumatology Department, University Hospital Reina Sofía, Córdoba, Spain; 16 Pediatric Rheumatology Unit, Virgen del Rocío University Hospital, Sevilla, Spain; 17 Rheumatology Department, Germans Trias i Pujol University Hospital, Badalona, Spain; 18 Rheumatology Department, Miguel Servet University Hospital, Zaragoza, Spain; 19 Pediatra. Pediatric Rheumatology Unit, Virgen de la Arrixaca University Clinical Hospital, Murcia, Spain; 20 Institut de Recerca Sant Joan de Déu, Universitat de Barcelona (UB), Barcelona, Spain

**Keywords:** Juvenile dermatomyositis, Clinical features, Medical tests, Prognostic factors

## Abstract

**Background:**

Juvenile Dermatomyositis (JDM) is the most common chronic idiopathic inflammatory myopathy in children. The diagnosis is clinical. Baseline laboratory and complementary studies trace the phenotype of these patients. The objective of this study was to describe epidemiological, clinical and laboratory characteristics at diagnosis of JDM patients included in the Spanish JDM registry, as well as to identify prognostic factors on these patients.

**Methods:**

We retrospectively reviewed clinical features, laboratory tests, and complementary studies at diagnosis of JDM patients included on the Spanish JDM registry. These data were analyzed to assess whether there was a relationship with the development of complications and time to disease inactivity.

**Results:**

One hundred and sixteen patients from 17 Spanish paediatric rheumatology centres were included, 76 girls (65%). Median age at diagnosis was 7.3 years (Interquartile range (IQR) 4.5–10.2). All patients had pathognomonic skin lesions at the beginning of the disease. Muscle weakness was present in 86.2%. Median Childhood Muscle Assessment Scale was 34 (IQR 22–47). Twelve patients (34%) had dysphagia and 3,5% dysphonia. Anti-p155 was the most frequently detected myositis specific antibody, followed by anti-MDA5. Twenty-nine patients developed calcinosis and 4 presented with macrophage activation syndrome. 70% reached inactivity in a median time of 8.9 months (IQR 4.5–34.8). 41% relapsed after a median time of 14.4 months (IQR 8.6–22.8) of inactivity. Shorter time to treatment was associated with better prognosis (Hazard ratio (HR) = 0.95 per month of evolution, *p* = 0.02). Heliotrope rash at diagnosis correlates with higher risk of development complications.

**Conclusions:**

We describe heliotrope rash as a risk factor for developing complications in our cohort of JDM patients, an easy-to-evaluate clinical sign that could help us to identify the group of patients we should monitor closely for this complication.

## Background

Juvenile Dermatomyositis (JDM) is the most common chronic idiopathic inflammatory myopathy in children (85%). It’s a systemic vasculopathy characterised by muscle and skin involvement. Diagnosis is clinical, with the identification of pathognomonic cutaneous rashes (Gottron’s papules, heliotrope rash), usually accompanied by proximal muscle weakness. Baseline laboratory and complementary studies trace the phenotype of these patients [1]. The goal of treatment is disease remission, reducing the development of complications, such as calcinosis. First line therapy includes systemic corticosteroids and subcutaneous methotrexate, adding intravenous immunoglobulins in selected patients. Although mortality remains below 4%, morbidity continues to be high (70–80%), predominantly on cutaneous, endocrine, muscular, and skeletal domains [1–17].

The objectives of this study were to describe epidemiological, clinical and laboratory findings at time of diagnosis of JDM patients included in the Spanish JDM registry, as well as to identify prognostic factors in these patients.

## Methods

### Study population and inclusion criteria

This is a retrospective descriptive observational and multicentre study of the JDM Spanish registry. Sant Joan de Déu Hospital (Barcelona) was the coordinator centre. Registration data were entered from January 2013 to January 2021. This study was approved by the Ethics committee with the code CEIC PIC-74-13. Inclusion criteria were diagnosis of JDM according to the Bohan and Peter criteria and/or expert diagnosis supported by Magnetic resonance image (MRI), electomyogram (EMG) or muscle biopsy evidence of myositis. Informed consent was obtained via the signature of the patient or their parent/legal guardian. Overlapping syndrome were not included in the registry.

### Data collection

Data collection was carried out retrospectively, reviewing data from the medical records until the last visit made at the centres of origin. Of the patients diagnosed before 2013, only those who still had a visit to the pediatric rheumatology unit between the period from 2013 to 2021 were included. We registered demographic (age, sex, concomitant medical history of immune-mediated pathology and family history of immune-mediated pathology, previous vaccines and infections to the onset of symptoms, age at disease onset, age at diagnosis, time to diagnosis since onset symptoms); clinical features at diagnosis like skin manifestations: Gottron’s papules, erythematous lesions, heliotrope rash, vasculitis lesions, subcutaneous oedema, atrophy, livedo reticularis, periungueal erythema, skin ulcers, shawl rash, poikiloderma, oral ulcers; weakness: pelvic girdle weakness, scapular girdle weakness and axial weakness; other symptoms: arthritis, constitutional symptoms (fever, weight loss, asthenia), gastrointestinal symptoms (dysphagia, abdominal pain, perforation bowel), pulmonary symptoms (dysphonia, interstitial lung disease (ILD), cardiovascular involvement and Raynaud’s phenomenon).

Laboratory tests collected were acute phase reactants: erythrocyte sedimentation rate (ESR) and C reactive protein (CRP); muscle enzymes: creatine phosphokinase (CK), aspartate aminotransferase (GOT), alanine aminotransferase (GPT), aldolase and lactate dehydrogenase (LDH), and myositis specific antibodies (MSA) and myositis associated antibodies (SMA) by immunoblot/blot-line. The reagent kit we used is Euroline Myositis Profile 16 from Euroimmun (Lübeck, Germany). Laboratory parameters were adjusted based on age-defined upper limits of normal. We used the Childhood Muscle Assessment Scale (CMAS) to assess muscle strength when applicable, we considered a normal score ≥ 48/52 points. Medical test evaluated: MRI and whole body MRI (WBMRI), EMG, muscle biopsy, video fluoroscopy/barium studies, echocardiography and electrocardiogram (ECG), pulmonary function tests and nailfold capillaroscopy.

We considered disease inactivity based on a modification of the Paediatric Rheumatology International Trials Organisation (PRINTO) criteria: absence of skin disease at the time of assessment, and at least 3 of the following 4 criteria: (1) creatine kinase (CK) ≤ 150 units/liter, (2) Childhood Myositis Assessment Scale (CMAS) score ≥ 48/52, (3) Manual Muscle Testing 8 (MMT-8) score ≥ 78/80, and (4) physician global assessment ≤ 0.2 (of a possible 10).

We registered time to inactivity disease and complications. Complications were calcinosis, macrophage activation syndrome (MAS), others included infections, osteoporosis and lipodystrophy. We analysed each clinical, laboratory and medical tests at diagnosis to detect prognostic factors that predispose to the development of complications and the time to reach inactivity. The times of disease inactivity and relapses were calculated retrospectively according to the status of the patients on the date of inclusion in the registry. We considered shorter time to reach remission and absence of complications as optimal treatment objectives.

### Statistical data analysis

We performed statistical analysis with SPSS 19.0 statistical software (Armonk, NY: IBM Corp). Descriptive statistics of the study variables with absolute frequency and percentage in the case of qualitative variables and median and interquartile range (IQR) for continuous variables. A verification of the study groups was carried out using the X test or Fisher’s exact test when necessary for qualitative variables. The comparison of the quantitative variables according to the study groups was carried out using the Student t-test for independent samples (or Welh test) or the MannWhitney U test (depending on whether or not they followed a normal distribution). The level of statistical significance was set at *p* < 0.05.

Survival analysis for each categorical variable (skin manifestations; type of weakness; other symptoms; laboratory tests; myositis specific antibodies and myositis associated antibodies; medical test evaluated) of interest was obtained by analysing the time until inactivity and complications, estimating the survival curve for each value of the variable using the Kaplan-Meier method and comparing them using the log-rank test. If the variable was numerical, we used Cox Proportional Hazards Regression.

## Results

### Demographic and clinical features

One hundred and sixteen patients from 17 Spanish paediatric rheumatology centres were included, 76 girls (65%). Of the 116 patients, 70 were diagnosed before 2013. Median age at diagnosis was 7.3 years (Interquartile range (IQR) 4.5–10.5). All patients had pathognomonic skin lesions at the beginning of the disease. Muscle weakness was present in 86.2% (108/116). Median Childhood Muscle Assessment Scale was 34 (IQR 22–47). 37% (43/116) of patients were associated with other symptoms at the beginning of the disease. 34% (12/116) were referred for dysphagia and 3.5% (4/116) dysphonia. Table 1 summarises demographic and clinical features. Median time to start treatment since the beginning of the disease was 2.5 moths (IQR 1.2–6.3), with systemic steroids as the most frequently used drug (98.3%), followed by methotrexate (76.7%) and hydroxychloroquine (53.4%).

**Table 1:** Demographic and clinical features

*1. Family and personal background*	
Concomitant medical history of immune-mediated pathology	8/116 (6.9%)
Family history of immune-mediated pathology	32/114 (28.1%)
Previous infection *	18/114 (15%)
Previous vaccination *	3/110 (2.7%)
Female	76/116 (65%)
Age at diagnosis (years; median and IQR)	7.3 (IQR 4.47–10.25)
Age at disease onset (years; median and IQR)	6.7 (IQR 4.00-9.64)
Time to diagnosis (moths; median and IQR)	2.7 (IQR 1.44–6.68)
*2. Clinical disease features*	
2.1 Skin manifestations	116/116 (100%)
• Gottron’s papules	104/116 (90.4%)
• Erythematous lesions	98/116 (84.5%)
• Heliotrope rash	75/116 (65.2%)
• Vasculitic lesions	39/116 (33.9%)
• Subcutaneous oedema	17/116 (14.6%)
• Atrophy	14/116 (12.1%)
• Livedo reticularis	7/116 (6%)
• Periungueal erythema	6/116 (5.2%)
• Skin ulcers	5/116 (4.3%)
• Shawl rash	3/116 (2.6%)
• Poikiloderma	3/116 (2.6%)
• Oral ulcers	2/116 (1.7%)
2.2 Weakness	108/116 (86.2%)
• Pelvic girdle weakness	90/108 (83.3%)
• Scapular girdle weakness	77/108 (71.3%)
• Axial weakness	69/103 (67%)
2.3 Other symptoms	43/116 (37%)
• Arthritis	17/116 (14.7%)
• Constitutional symptoms (fever, weight loss, asthenia)	11/116 (9.5%)
• Gastrointestinal symptoms:	
◦ Dysphagia	12/116 (10.3%)
◦ Abdominal pain	7/116 (6%)
◦ Perforation bowel	1/116 (0.9%)
• Pulmonary symptoms:	
◦ Dysphonia	4/116 (3.5%)
◦ Interstitial lung disease (ILD)	1/116 (0.9%)
◦• Cardiovascular involvement	2/116 (1.7%)
◦• Raynaud’s phenomenon	2/116 (1.7%)
3. *Complications*	35/116 (30.2%)
◦• Calcinosis	29 (25%)
◦• Macrophage activation syndrome	4 (3.5%)
◦• Osteoporosis	3 (2.6%)
◦• Lipodystrophy	2 (1.7%)
◦• Infections	2 (1.7%)
*4.* *CMAS* (*Childhood Muscle Assessment Scale) (N = 47)*	Median = 34 points IQR = 22–47 points

*In the three-period-month before diagnosis

### Laboratory parameters and diagnostic features

#### Laboratory parameters

Forty-two and 52.9% of patients had erythrocyte sedimentation rate (ESR) and C reactive protein (CRP) increased. According to muscular enzymes, aldolase was the most frequently raised (78%), followed by alanine aminotransferase (GPT) 75%, aspartate aminotransferase (GOT) 66%, lactate dehydrogenase (LDH) 56.5%, and creatine phosphokinase (CK) 52%.

We identified a myositis specific antibody (MSA) in 27.3% of patients (21/77), with anti-p155 as the most frequently detected (12.8%), followed by anti-MDA5 (11.1%). Table 2 describes laboratory parameters.

**Table 2:** Laboratory parameters

Variable (number of patients)	Median	IQR	Reference range
*2.1 Acute phase reactants*			
Erythrocyte sedimentation rate (mm/h) (*N* = 87)	13.0	4.0–24.0	0–15 mm/h
C reactive protein (mg/L) (*N* = 85)	1.9	0.4-5.0	0–5 mg/L
*2.2 Muscle enzymes*			
Creatine phosphokinase (U/ml) (*N* = 110)	281.0	90.8–3175	0-200 U/ml
Aspartate aminotransferase (U/L) (*N* = 113)	84.0	36.5-172.5	0–35 U/I
Alanine aminotransferase (U/L) (*N* = 112)	61.0	25.3–151	10–40 U/I
Aldolase (U/L) (*N* = 77)	13.1	8.1–26.3	0-7.9 U/l
Lactate dehydrogenase (U/L) (*N* = 108)	618.0	450.8-1112.3	120–300 UI/l
*2.3 Myositis specific antibodies Sample and percentage*			
Anti p155 Anti MDA5 Anti NXP2 Anti Mi2 Anti SRP Anti PL12	6/47 (12.8%) 4/36 (11.1%) 4/46 (8.7%) 4/65 (6.2%) 2/42 (4.8%) 1/41 (2.4%)		
*2.4 Myositis associated antibodies (SMA) Sample and percentage*			
Anti Ro52 Anti PM Scl	3/37 (8.1%) 3/44 (6.8%)		

#### Diagnostic features

Magnetic resonance imaging was obtained in 73 patients at diagnosis. As expected, pelvic girdle muscles were the muscular group most frequently involved (79%). Whole-body MRI (WBMRI) was even more sensitive, detecting muscle inflammation in 94% of the patients studied.

Only 17 patients (14.7%) had video fluoroscopy/Barium studies, 16 of them asymptomatic from a gastrointestinal standpoint. We detected swallowing dysfunction in 9/16 (56.3%) asymptomatic patients.

At diagnosis twenty-seven of 74 patients (35.1%), had pulmonary function tests (PFTs) if feasible due to age. Four patients (15.4%) presented abnormal results (3 patients with restrictive pattern, 1 obstructive pattern). None of these four patients had interstitial lung disease (ILD) related with JDM on high resolution CT. According to MSA, we performed PFTs in two of the four MDA5 + patients. Both were normal.

Muscle biopsy at diagnosis was performed in 66 patients (56.9%). Ninety-six per cent (63/65) of patients had abnormal microscopic findings. The two patients with a muscle biopsy reported as normal, had classic JDM symptoms, including muscle involvement documented by physical examination and/or other complementary tests. Table 3 describes diagnostic features.

**Table 3:** Diagnostic features

Medical test	Abnormal result
MRI (*N* = 73) • Pelvic girdle oedema • Scapular girdle oedema • Subcutaneous cellular tissue oedema • Fascia involvement Whole body MRI (WBMRI) (*N* = 34)	63/73 (86.3%) 53/67 (79.1%) 45/61(73.8%) 34/71 (47.9%) 27/71 (38%) 32/34 (94.1%)
Electromyogram EMG (*N* = 94)	83/94 87.4%
Muscle biopsy (*N* = 65) • Incomplete study of muscle biopsy	63/65 (96.9%) 1/66 (1.5%)
Video fluoroscopy/Barium studies (*N* = 17)	10/17 (58.8%)
Echocardiography and electrocardiogram ECG (*N* = 74)	2/74 (2.7%)
Pulmonary function tests (*N* = 27)	4/27 (15.4%)
Nailfold capillaroscopy (*N* = 49)	44/49 (89.8%)

### Disease course

Sixty-one patients (52.6%) achieved inactivity until the last follow-up visit registered. The median time to achieve inactivity was 8.9 months (IQR 4.5–34.8). Seventy patients were diagnosed before opening the registry. Laboratory parameters normalized 1.4 months before reaching clinical inactivity, with skin disease as the latest to be controlled (median 8.0 months (IQR 3.5–30.0). Twenty-nine (25%) developed calcinosis and four patients (3.4%) presented MAS. Forty-one per cent (37/90) relapsed after 14.4 months of inactivity (IQR 8.6–22.8). The number of each relapse was not recorded, only the first. The denominator 90 refers to the total number of patients in whom the relapse variable could be analyzed; in the remaining 26, the data was not collected.

### Prognostic factors

Clinical, laboratory, and medical tests at diagnosis were analysed to detect prognostic factors. The longer the time to start treatment, the lower the probability of reaching inactivity disease (Hazard ratio (HR) 0.95 per month of treatment delay, *p* = 0.02). The presence of heliotrope rash at diagnosis was associated with a higher risk of development of complications (Fisher test 0,009; OR 0.35, IC 95% 0,14-0.83) with a close statistical significance for the development of calcinosis (Fisher test 0,07; OR 0.33, IC 95% 0,10-1.06). We cannot calculate the survival curve for the time until the onset of calcinosis because the date of the calcinosis event was not recorded. We did not detect any variable that could help us to predict MAS.

## Discussion

To date, this is the largest multicentre cohort of JDM patients in our country. Patient characteristics are similar to previously reported series, with a female predominance (65%) 18–20, 24–33, but an older mean age at diagnosis compared with the CARRA cohort 19, 20. Cutaneous disease was the first organ involved, this could explain the shorter time to diagnosis compared with the British cohort 18, 21. 37% of patients complained about other symptoms at diagnosis apart from cutaneous and muscular disease, with arthritis as the most frequently described symptom (14.7%). We would like to remark this data because according to our experience, in some cases there is a misdiagnosis with juvenile idiopathic arthritis, mainly in JDM anti-MDA5 + patients, with the consequent delay in completing the basal study and starting the adequate immunosuppression for JDM.

According to laboratory tests, CK was normal in 48% of patients, with aldolase as the most frequently increased (78%) despite being the less frequently ordered. EMG and MR increased the probability of detecting muscle involvement (87.40 and 86.3% respectively). Sensitivity increased to 95% when we assessed WBMRI. All but one patient who had a muscle biopsy performed had pathological results, supporting the importance of taking biopsy in case of diagnostic uncertainty [10].

A MSA was identified in 27.3% of patients, a lower percentage compared to other series [18–22], explained because we are in front of a retrospective study and not all the MSA were performed in each patient. The most frequently MSA detected was anti-p155 (12.8%), followed by anti-MDA5 (11.1%) and anti-NXP2 (8.7%), similar to previously published [2–4].

At the time of enrolment to the registry, there was no consensus about performing high-resolution CT pulmonary scan at diagnosis as part of the baseline study except in case of pathological PFTs. We detected ILD in one of the four MDA5 + patients when she altered PFTs after 3.6 years of JDM diagnosis.

Despite just 34% (12/116) referred for dysphagia, swallowing disturbances were presented in 56.3% (9/16) asymptomatic patients. This result emphasizes the importance of performing a complete evaluation of dysphagia, not limited to anamnesis, especially considering the implication of swallowing impairment for patient management.

Calcinosis was present in 10.3% of patients (12/116) at diagnosis, with an increase to 25% if we consider all the patients with calcinosis at the time of being included in the registry (29 patients, median of follow up of 59 months since diagnosis (IQR 16.25–109). Only four patients were anti-NXP2+, therefore we could not evaluate if there was a higher risk of calcinosis on these patients in our series. Heliotrope rash at diagnosis was related with a higher risk of development of complications, Moreover, it was close to statistical significance for the development of calcinosis. To the best of our knowledge, this is the first time that a clinical variable, as easy to assess as heliotrope rash, can be related to complications like calcinosis and it raises the question of whether in patients with heliotrope we should perform a low-radiation total body CT to detect calcinosis at JDM diagnosis. In others publications like of Nozawa et al. described nailfold capillary changes as predictors of calcinosis in JDM [23].

Unfortunately, we did not identify any prognostic factor to recognize patients with higher risk of developing MAS. Despite MAS being a rare JDM complication, 3.4% of our cohort presented it in the three-month period after diagnosis, and due its severity it must be considered and actively searched. Moreover, awareness of this complication is important because elevation of AST and/or ALT could be interpreted as muscular origin causing a delay on MAS diagnosis.

41% of patients relapsed after a median time of 14.4 months of inactivity underscoring the need for continued regular follow-up. We would also like to highlight the importance of new biomarkers, such as plasma interferon signature or circulating endothelial cells that allow us to detect a relapse prior to clinical or analytical findings [2–4]. We have no data of these biomarkers in our cohort but could be interesting to study in future studies.

We find some limitations in our study, most of them due to being a retrospective study. Another important limitation was that of the patients diagnosed before 2013, only those who still had a visit to the pediatric rheumatology unit between the period from 2013 to 2021 were included. Furthermore, another limitation is the low percentage of patients in whom SMA were performed. Recent publications, of Papadopoulou et al. and McCann et al. have described how having an SMA modifies a patient´s prognosis, as it is the case of anti-MDA5 patients with a higher risk of ILD.

## Conclusions

In conclusion, we present data from the JDM Spanish registry and identify heliotrope rash as a risk factor in the development of complications in JDM patients. This easy-to-evaluate clinical sign could help us to identify a subgroup of patients with higher risk whom we should monitor more closely. We need larger registries, preferably prospective, that can confirm these findings.

## Data Availability

The datasets used and/or analysed during the current study are available from the corresponding author on reasonable request.

## Abbreviations

JDM: Juvenile Dermatomyositis
CMAS: Childhood Muscle Assessment Scale
MAS: Macrophage activation syndrome
IQR: Interquartile range
N: Sample
ILD: Interstitial lung disease
ESR: Erythrocyte sedimentation rate
CRP: C reactive protein
GPT: Alanine aminotransferase
GOT: Aspartate aminotransferase
LDH: Lactate dehydrogenase
CK: Creatine phosphokinase
MSA: Myositis specific antibody
SMA: Myositis associated antibodies
MRI: Magnetic resonance image
WBMRI: Whole-body MRI
PFTs: Pulmonary function tests
CT: Computed axial tomography
EMG: Electromyogram
ECG: Electrocardiogram
HR: Hazard ratio
